# Understanding Attorneys’ Plea Advice: The Role of Defendant Guilt and Trial Penalties

**DOI:** 10.3390/bs15111465

**Published:** 2025-10-28

**Authors:** Janice L. Burke, Miko M. Wilford, Yueran Yang

**Affiliations:** 1 JurySync LLC, Olathe, KS 66061, USA; 2 Department of Psychology, Iowa State University, Ames, IA 50011, USA; mwilford@iastate.edu; 3 Department of Psychology, University of Nevada, Reno, Reno, NV 89557, USA; yuerany@unr.edu

**Keywords:** plea bargaining, attorneys, recommendation, defendant guilt, trial sentence

## Abstract

Plea bargaining underlies the majority of criminal convictions in the United States, yet concerns remain about its potentially coercive effects, particularly when sentencing differentials between plea offers and potential trial outcomes are large. This experiment examined practicing attorneys’ plea-related recommendations in a 2 (Defendant guilt status: guilty or innocent) × 3 (Potential trial sentence: low, moderate, or high) between-subjects design. Using an interactive computer simulation designed to convey legal scenarios engagingly, we measured attorneys’ plea recommendations, willingness to recommend the plea (WTRP), and maximum acceptable plea sentences. The results reflected Prospect Theory’s utility function, with plea acceptance recommendations increasing as potential trial sentences increased, provided the plea sentence remained within an acceptable range. Attorneys also accepted longer maximum plea sentences as trial penalties became more severe. An interaction between defendant guilt status and potential trial sentence showed that attorneys wanted shorter maximum plea sentences for innocent defendants, though this effect was moderated by trial sentence severity. These findings contribute to our understanding of how attorneys evaluate plea offers and illustrate how large sentencing differentials can shape their recommendations in ways that may affect the fairness of the plea-bargaining process.

## 1. Introduction

Most criminal convictions in the United States are the result of guilty pleas, many of which are a product of the “bargaining” process (Dervan, 2019). Some researchers and legal scholars argue that plea bargaining can be coercive for defendants because the sentence discrepancies between plea offers and potential trial sentences can be massive (Wilford & Bornstein, 2023), seemingly penalizing defendants for exercising their constitutional right to trial (McCoy, 2005). Consequently, innocent defendants can be enticed with large plea discounts (or threatened with substantial trial penalties), leading them to be wrongfully convicted via their own false guilty pleas (e.g., Schneider & Zottoli, 2019). Indeed, a large proportion (24%) of known exonerees falsely pleaded guilty (National Registry of Exonerations, 2024). The issue concerning these sentencing discrepancies is partially rooted in the Supreme Court’s legal precedent for plea bargaining. In *Brady v. United States* (1970), the Court ruled that a guilty plea is not rendered involuntary just because a defendant pleaded guilty to secure a certain and more lenient outcome rather than risk a harsher sentence at trial. While the courts have solidified plea bargaining as a constitutionally sound practice, they have also recognized the importance of counsel in preserving the validity of plea convictions (e.g., *Lafler v. Cooper*, 2012; *Jae Lee v. United States*, 2017).

### 1.1. Attorney Influence in Plea Decisions

Criminal defendants often face plea decisions with limited information and under considerable pressure, making them heavily reliant on their attorneys for guidance. Vignette and experimental studies have consistently shown that attorneys’ recommendations significantly influence defendants’ plea decision-making (e.g., Henderson & Levett, 2018, 2019; Henderson & Shteynberg, 2020; Henderson et al., 2023). For instance, Henderson and Levett (2018) adapted a cheating paradigm in which participants were accused of academic dishonesty and offered a plea bargain. They were given the option to admit guilt and accept a lab-imposed punishment (serving as the plea offer) or to contest the accusation and wait for a simulated academic conduct hearing (analogous to a trial). Before deciding, participants received advice from an advocate who either recommended accepting the plea, advised going to trial, or neutrally explained both options without making a recommendation. The findings showed that participants’ choices were strongly shaped by the advocate’s guidance, with innocent participants proving particularly susceptible to such influence. Those who were encouraged to go to trial were far less likely to plead guilty than participants in any other condition, illustrating how attorney advice can be a decisive factor in defendants’ plea decisions (Henderson & Levett, 2018). Other vignette-based work, such as Lee et al. (2021), reinforced that defendants’ plea decisions are highly susceptible to attorney recommendations, finding that participants overwhelmingly followed the advice of counsel even when it conflicted with their own case assessments. Similarly, Frazier and Gonzales (2022) found that defendants’ self-perceived guilt influenced how they interpreted and weighed attorney advice, consistent with evidence that innocent participants may be particularly vulnerable to influence.

Drawing on interviews and surveys with practicing attorneys, Suiter and Metcalfe (2024) examined how attorneys assess case risks, manage client expectations, and navigate ethical considerations during plea negotiations. They found that attorneys’ professional experiences and perceptions of potential trial outcomes shaped the guidance they provided, which in turn influenced defendants’ plea decisions. More broadly, Wright and Roberts (2023) reviewed attorneys’ role in shaping plea negotiations, highlighting how professional norms and institutional pressures frame the advice attorneys provide. Other surveys of attorneys have further emphasized the complexity of their role, especially given constrained resources (e.g., Helm et al., 2018) and clients with significant legal misconceptions (e.g., Wilford et al., 2025). Relatedly, Shook et al. (2021) demonstrated in a juvenile context that defense attorneys influence not only plea outcomes but also defendants’ perceptions of court legitimacy (see Viljoen et al., 2005, for additional information on the influence attorneys have on juvenile defendants). This growing body of work underscores the importance of uncovering what motivates attorneys’ plea-related recommendations.

### 1.2. Shadow of the Trial (SoT) Model

Plea decisions have long been modeled in the Shadow of the Trial (SoT) (Mnookin & Kornhauser, 1979). SoT posits that plea decisions generally reflect a comparison of expected trial outcomes and plea offers, typically conceptualized as follows:

$$Decision\;weight=\left[Trial\;conviction\;probability\times Trial\;sentence\right]-Plea\;sentence$$

When the decision weight is a positive value (indicating that trial is expected to yield a worse outcome than the plea offer), defendants are expected to accept the plea. When the weight is negative (indicating that the plea offer is worse than the expected trial outcome), defendants should reject the plea offer and proceed to trial (Bibas, 2004). Thus, the conviction probability at trial, potential trial sentence, and plea sentence are generally expected to have predictive effects on plea decisions.

Several studies have directly tested SoT factors among courtroom actors (e.g., Bushway & Redlich, 2012; Bushway et al., 2014). For example, Bushway and colleagues (Bushway et al., 2014) surveyed prosecutors, defense attorneys, and judges and found that the conviction probability (based on evidence strength), potential trial sentence, and plea offer were the primary predictors of plea outcomes. Wilford et al. (2021a) recently expanded the model to incorporate defendants’ guilt status. Accordingly, an updated representation of SoT now identifies conviction probability, potential trial sentence, plea offer, and defendant guilt status as the primary predictors of plea decisions.

Subsequent studies have further supported the influence of SoT factors on attorneys’ plea recommendations (Cardenas, 2023; Fessinger et al., 2024; Hellgren & Kassin, 2022; Henderson, 2021; Kramer et al., 2007). For instance, Cardenas (2023) observed that when the potential trial sentence was high, attorneys were more likely to recommend plea acceptance and defendants were subsequently more likely to accept the plea. Fessinger et al. (2024) similarly found that attorneys were more likely to recommend plea acceptance when defendants were guilty and facing harsher potential trial sentences. Henderson (2021) found that strong evidence increased the likelihood that attorneys would recommend plea acceptance. Kramer et al. (2007) tested all SoT factors simultaneously (along with defendants’ preferences for trial) and found that attorneys were most likely to recommend plea acceptance when the potential trial sentence was high, evidence was strong, and defendants indicated a preference for trial. Similarly, Hellgren and Kassin (2022) found that conviction probability—rather than defendant claims of guilt or innocence—was the strongest predictor of attorneys’ plea recommendations, further supporting the centrality of SoT factors in attorney decision-making.

Edkins (2011) similarly examined attorneys’ plea recommendations and found that these recommendations were influenced by the evidence strength and potential trial sentence—two core components of the SoT framework. Importantly, Edkins (2011) also observed disparities in plea recommendations by defendant race, which were not explained by differences in attorneys’ perceptions of conviction probability or potential trial sentence, suggesting that the SoT factors alone could not fully account for differences in attorneys’ recommendations.

While SoT provides a robust framework for predicting plea outcomes at the aggregate level, prior work has noted its limitations at the individual level. Specifically, researchers have argued that variability in plea decisions may reflect individual differences (e.g., risk preferences, implicit biases, etc.; Bushway & Redlich, 2012; Bushway et al., 2014; Edkins & Dervan, 2018) and contextual factors (e.g., case characteristics, defendant criminal histories, etc.; Greenberg, 2021; Votruba & Tisdale, 2021) that SoT does not incorporate. In the context of plea decisions, attorneys may weigh the same conviction probability and potential trial sentence differently depending on their risk-aversion or risk-seeking tendencies. This perspective highlights the need to investigate how attorneys’ plea decision-making relates to their risk attitudes across different case factors, as such differences may account for variability that the traditional SoT model leaves unexplained.

### 1.3. Prospect Theory (PT)

Because the plea-bargaining process inherently involves risk and uncertainty, researchers have increasingly turned to Prospect Theory (PT) to better understand the factors that influence decision-making (Bartlett & Zottoli, 2021; Edkins & Dervan, 2018; Garnier-Dykstra & Wilson, 2021). PT is a foundational theory from behavioral economics that predicts people evaluate potential outcomes relative to a reference point (e.g., the status quo or expected baseline), and that this reference point shapes their choices under uncertainty (Kahneman & Tversky, 1979). A central component of PT is the value (utility) function, which is S-shaped: concave for gains and convex for losses, with gains and losses being determined relative to the reference point (Tversky & Kahneman, 1992). This S-shape reflects differing risk attitudes in the two contexts (Kahneman & Tversky, 1979; Barberis, 2013). That is, people are typically risk-averse when facing potential gains, preferring a sure gain to a gamble. In contrast, they are typically risk-seeking when facing potential losses, preferring to take a gamble if they can avoid a sure loss. These risk attitudes are critical to understanding decisions made under uncertainty.

Empirical research has begun to explore how components of PT map onto plea decision-making, offering support for the idea that risk attitudes play a meaningful role (Bartlett & Zottoli, 2021; Edkins & Dervan, 2018; Garnier-Dykstra & Wilson, 2021). For example, Edkins and Dervan (2018) found that defendants were more likely to accept plea offers when guilty and more likely to reject them when innocent, even when the potential consequences of trial were severe. This pattern of results supported the notion that innocent defendants are more likely to perceive a plea as conferring a loss (i.e., any punishment is a loss because they did not do anything wrong), while guilty defendants are more likely to perceive it as conferring a gain (i.e., a discount on the punishment they would otherwise receive; Redlich et al., 2017). Garnier-Dykstra and Wilson (2021) extended this work by demonstrating that defendants’ risk preferences significantly influenced their plea decisions, particularly as the disparity between plea and trial sentences increased. In a broader examination, Bartlett and Zottoli (2021) observed that defendants’ plea decisions were shaped by perceived case strength and the framing of outcomes as gains or losses, consistent with PT’s predictions. Together, this emerging body of work underscores the relevance of PT for understanding the psychological processes underlying plea outcomes and sets the stage for examining how attorneys apply these elements to their own decision-making.

Extending this line of research, PT offers a compelling framework for predicting how attorneys might evaluate plea offers in light of the defendant’s factual guilt or innocence and trial risks. Previous research has shown that attorneys’ decisions and perceptions vary depending on the perceived guilt of their clients (Redlich et al., 2010). Thus, defendant guilt status likely serves as a key reference point shaping how attorneys interpret the plea-trial tradeoff. When the defendant is guilty, the plea offer may be viewed as a relative gain (i.e., avoiding a harsher sentence), prompting risk-averse behavior and manifesting as a greater likelihood of recommending plea acceptance. As the potential trial sentence increases, the appeal of this “gain” also increases. In contrast, when the defendant is innocent, the plea may be seen as a relative loss (i.e., accepting punishment despite the potential for acquittal), leading to risk-seeking behavior and manifesting as a greater inclination to recommend rejecting the plea. However, this preference may weaken as the potential trial sentence becomes more severe, shifting the perception of what is at stake. These shifting dynamics suggest that attorneys’ plea recommendations could be shaped not only by the magnitude of sentencing outcomes but also by how defendant guilt status influences their risk preferences.

## 2. Research Overview

The present experiment examined how attorneys’ plea recommendations are influenced by defendant guilt status and potential trial sentence. Although Hellgren and Kassin (2022) found that defendants’ claims of guilt or innocence had little effect on attorneys’ plea recommendations, we expected a different pattern when factual guilt or innocence was introduced. Applying PT’s utility function, we posited that defendant guilt status could serve as a reference point that alters how attorneys evaluate the plea-trial tradeoff (Redlich et al., 2017). When the defendant is guilty, the plea offer could be viewed as a relative gain, activating the concave portion of the utility function and prompting risk-averse behavior. When the defendant is innocent, the plea could be viewed as a relative loss, placing attorneys in the convex portion of the utility function and encouraging risk-seeking behavior. Thus, we hypothesized that participants would be more likely to recommend plea acceptance when the defendant was guilty than when the defendant was innocent (Hypothesis #1).

Previous research suggests that attorneys’ plea recommendations are sensitive to the magnitude of the potential trial sentence (e.g., Cardenas, 2023; Wilford et al., 2021b). According to PT’s utility function, larger potential losses (e.g., longer potential trial sentences) shift the subjective value of the plea offer, making the relative gain of accepting the plea more appealing. In this context, the escalating cost of trial should enhance risk aversion and increase the likelihood of recommending plea acceptance. Thus, we hypothesized that participants would be more likely to recommend plea acceptance in the high potential trial sentence conditions than in the moderate or low potential trial sentence conditions (Hypothesis #2).

We also predicted that the influence of potential trial sentence on attorneys’ plea recommendations would differ depending on defendant guilt status. According to PT’s utility function, people tend to be risk-averse when facing potential gains and risk-seeking when facing potential losses (Kahneman & Tversky, 1979; Barberis, 2013). As previously discussed, we expected participants to be more likely to recommend a plea deal (i.e., be risk-averse) when defendants are guilty, and we expected participants to be more likely to recommend rejecting a plea deal (i.e., be risk-seeking) when defendants are innocent. Consequently, high potential trial sentences would be expected to enhance any risk aversion already present (when defendants are guilty); in contrast, the trial sentence must be relatively large to overcome an initial risk-seeking preference (when defendants are innocent). Thus, we hypothesized an interaction between defendant guilt status and potential trial sentence, such that the effect of potential trial sentence on plea recommendations would be more pronounced for guilty than for innocent defendants (Hypothesis #3). We expected the same patterns from Hypotheses #1 through #3 to emerge for participants’ willingness to recommend the plea (WTRP; 0–100%) and the maximum plea sentences they would accept (years/months).

## 3. Method

To assess and refine the study method, we conducted a pilot test with a sample of legal professionals (*n* = 31) drawn from the authors’ professional networks. The primary goal was to ensure that the experimental manipulations of defendant guilt status and potential trial sentence severity were effective. The guilt status manipulation focused on factual guilt, such that each scenario explicitly indicated whether the defendant had disclosed their guilt or innocence to their attorney. In each scenario, additional narrative details were included to substantiate the defendant’s claim. Pilot participants were asked to categorize each scenario as reflecting a guilty or innocent defendant. All participants correctly categorized the guilt status conditions, confirming efficacy of the manipulation.

The pilot also tested the potential trial sentence manipulation, which included three levels of severity: 2 years (low), 4 years (moderate), and 20 years (high). These sentence lengths were based on Nevada’s sentencing guidelines for drug possession, which is typically classified as a Category E felony and carries a penalty of 1 to 4 years in state prison (Nev. Rev. Stat. § 193.130, 2025). The 20-year condition was designed to reflect an overcharging scenario, in which prosecutors charge a more serious offense than the evidence may support to pressure defendants into accepting a plea deal (Bibas, 2004). Pilot participants were asked to evaluate whether each trial sentence condition represented a reasonable, moderate, or excessive punishment (2 years: 75% reasonable, 25% moderate; 4 years: 80% moderate, 20% excessive; 20 years: 100% excessive). In addition, we measured the proportion of participants who recommended plea acceptance under each condition (2-year: 25%; 4-year: 50%; 20-year: 57%), which allowed us to confirm that the materials elicited expected pattern of responses across sentencing levels. Based on this feedback, minor wording adjustments were made to improve clarity before launching the study.

### 3.1. Participants

Based on a power analysis for logistic regression to detect an odds ratio (OR) of two (i.e., effect size), we needed to recruit 400 practicing attorneys in the United States to achieve 95% statistical power. Our participant recruitment strategy was twofold: We recruited via CloudResearch’s Connect and via a snowball sampling strategy similar to Bushway and colleagues (Bushway et al., 2014). The combined final sample size was *N* = 403. See Table A1 in Appendix A for an overview of descriptive statistics for the entire sample. In the following sections, we summarize the recruitment procedures and key demographic characteristics for each sample.

#### 3.1.1. CloudResearch Sample

We recruited 337 participants from Connect, an online source of high-quality data by CloudResearch (CR). In the Connect platform, we included prescreening questions verifying that participants worked in the legal industry and held a law degree. In the survey itself, we confirmed that participants were practicing attorneys and followed up by asking what type of law they practice. Participants who did not meet the study criteria were screened out prior to beginning the study.

The sample was largely female (64.99%) and White (65.88%) with a mean age of 39.63 years (*SD* = 12.76), which generally aligns with the demographic composition of attorneys nationwide (American Bar Association, 2020). Participants worked primarily in densely populated (20.47%), urban jurisdictions (53.71%) from the southern region (40.95%) of the United States. Upon completion of the survey, each participant was compensated $10 for their participation via the Connect platform.

#### 3.1.2. Snowball Sample

We recruited 66 participants using a snowball strategy (Bushway et al., 2014). Specifically, we emailed state and national criminal defense attorney associations (e.g., Association of Criminal Defense Lawyers in Nevada, National Association for Public Defense) and public defenders’ offices. We contacted 50 organizations and asked them to send the survey invitation to their members. We sent up to two messages to each organization via their contact form or the email address listed on their website. Three of these organizations (6%) agreed to send the survey to their members-only listservs. We also reached out to personal contacts of the authors and posted on social media channels (e.g., Facebook, LinkedIn). Lastly, the survey included prompts on the first and final screens requesting that participants forward the survey invitation to other attorneys in their personal and professional networks.

The sample was roughly equally split by gender, with 48.48% reporting as female. Most participants were White (78.79%) with a mean age of 39.61 years (*SD* = 11.78), again generally aligning with the demographic composition of attorneys nationwide (American Bar Association, 2020). Participants worked mostly in moderately populated (24.24%), urban jurisdictions (59.09%) from the western region (51.52%) of the United States. Upon completion of the survey, each participant was compensated $10 for their participation via Tremendous, wherein they chose between an electronic gift card or a donation to pre-identified charities (e.g., NACDL Foundation for Criminal Justice, National Bail Fund Network, etc.).

### 3.2. Design

The experiment used a 2 (Defendant guilt status: guilty or innocent) × 3 (Potential trial sentence: low, moderate, or high) between-subjects factorial design. Participants were randomly assigned to one of the six conditions. Specifically, participants were informed whether the defendant was guilty or innocent (coded as 0 = guilty, 1 = innocent) and presented with a potential trial sentence of either 2, 4, or 20 years (coded as 0 = low, 1 = moderate, 2 = high). The primary dependent variable was a binary recommendation regarding whether their client should accept or reject a plea offer that conferred a 50% reduction to the trial sentence (coded as 0 = reject, 1 = accept). To capture more nuanced preferences, we also included a continuous measure of willingness to recommend the plea (WTRP), rated from 0 to 100%. While the binary item reflects a realistic decision point attorneys may face, the continuous scale offers a more sensitive assessment of attorneys’ openness to a plea, allowing us to detect more subtle differences in judgment. A second continuous dependent variable measured the maximum plea sentence participants would consider acceptable for their client to face, reported in years and months. Thus, the three dependent variables of interest were: participants’ plea recommendations (accept or reject), their willingness to recommend the plea (WTRP; 0–100%), and their maximum acceptable plea sentence (years/months).

### 3.3. Materials

The materials for this experiment included an interactive computer simulation (e.g., Wilford et al., 2021a), decision questionnaire, and demographics questionnaire (presented in that order). To simulate a plea-bargaining scenario, participants watched a series of three animated segments from a first-person point of view. In the first segment, participants watched a male avatar at a party with a female friend. Both people are approached by a drug dealer before the party is broken up by law enforcement. The male avatar flees to his car, where he is found by a law enforcement officer in possession of his female friend’s bag and drugs are spotted in plain sight. In the second segment, participants are approached by the same male avatar from the previous segment (i.e., the defendant). The defendant first informs participants that he is being charged with drug possession and asks them to serve as the defense attorney in this case. The defendant then informs participants whether they were guilty or innocent, and a flashback sequence is presented as objective evidence to corroborate his claim. The flashback definitively shows the defendant either accepting or rejecting drugs from the drug dealer. In the third segment, participants are approached by another avatar who introduces himself as the prosecuting attorney and informs them of the potential trial sentence (2, 4, or 20 years) and plea offer (carrying a sentence of 1, 2, or 10 years, respectively). See Appendix B for a script of the simulation.

The decision questionnaire first asked participants for their plea recommendation (accept or reject), followed by WTRP (0–100%) and maximum acceptable plea sentences (years/months). Next, participants responded to a series of questions intended to assess their values when making plea recommendations. Specifically, they were asked to rate the importance of several case-related factors, including: (1) conviction probability, (2) sentence length at trial, (3) sentence length of plea deal, and (4) defendant’s guilt or innocence. Each factor was rated on a 7-point Likert scale ranging from 1 (Not at all important) to 7 (Extremely important), with 4 representing a neutral midpoint. Lastly, the decision questionnaire included attention and manipulation checks, such as “*What crime was the defendant charged with?*” and “*What was the potential trial sentence?*”

The demographics questionnaire asked participants for their age, gender identity, and race/ethnicity. It also asked participants questions regarding their jurisdictions, including region, population, and density. Finally, it asked participants an open-ended question about their perceptions of plea bargaining.

### 3.4. Procedure

Participants received study invitations via email or CR Connect. The invitations included information regarding the study and a link to access it. First, the link directed participants to a Qualtrics survey in which they received the informed consent and instructions. Second, participants were directed to the PleaJustice webpage (pleajustice.org) where they entered the simulation and proceeded through the three animated segments. Third, participants were redirected back to the Qualtrics survey to complete the decision and demographic questionnaires. Because these specialized samples of attorneys are difficult and expensive to recruit, we collected data for this study concurrently with a second study, which used a different simulation. Participants completed both studies within the same session for efficiency. The second study’s simulation included its own set of questions. The simulations were counterbalanced such that the order in which participants received each was randomized, with one half completing the simulations in one order and the other half completing them in the reverse order. The survey included a total of 38 questions, and the average amount of time required to complete the study was approximately 15 min. Upon completion, participants were debriefed, thanked, and compensated for their participation.

## 4. Results

All analyses were performed using *R* software (v4.1.2; R Core Team, 2021). Prior to the main analyses, we performed preliminary checks to ensure data quality and model validity. We first examined data missingness and found no missing responses, rendering imputation unnecessary. We then analyzed responses to the attention and manipulation check questions. Most participants (99%) answered both questions correctly. Analyses were conducted both with and without the small number of participants who failed one or both checks. Because the results did not differ meaningfully, we report findings based on the full dataset. Next, we inspected descriptive statistics and histograms for the continuous dependent variables, including willingness to recommend plea (WTRP) and maximum acceptable plea sentences. Both variables showed evidence of positive skew (WTRP: skewness = 0.82, kurtosis = −1.40; max plea: skewness = 1.16, kurtosis = −2.00). We applied transformations and removed outliers (*n* = 16), and the primary findings remained consistent following these adjustments. Therefore, we retained the original variables for ease of understanding.

Assumption checks were then conducted for each model. For plea recommendations, we used logistic regression. Because predictors were categorical and the design was fully crossed, neither linearity nor multicollinearity was a concern. Sixteen potential outliers (4%) were flagged, but analyses with and without them yielded the same results, so all cases were retained. For the linear regression models (WTRP and maximum plea sentences), most assumptions were met. WTRP showed minor non-normality, and maximum plea sentences violated normality and homoscedasticity. Transformations, outlier removal, and robustness checks with parametric and non-parametric tests confirmed that results were consistent. Thus, we present findings from the original models for clarity and power. In the following sections, we discuss the findings for plea recommendations, WTRP, and maximum plea sentences.

### 4.1. Plea Recommendations

To test participants’ plea recommendations, we first explored descriptive statistics. Overall, participants were more likely to recommend that their client reject (59%) than accept (41%) the plea offer. Among those who recommended plea acceptance, 23% advised their client to falsely plead guilty. When examining defendant guilt status, 62% of participants recommended plea acceptance in the guilty defendant conditions, compared to only 23% in the innocent defendant conditions. Regarding potential trial sentence, plea acceptance rates were 47% in the low sentence conditions, 49% in the moderate sentence conditions, and 30% in the high sentence conditions.

Next, we tested Hypotheses #1–3 with a logistic regression model. The model included defendant guilt status, potential trial sentence, and their interaction term as predictor variables. The dichotomous outcome variable was participants’ plea recommendations (accept or reject). The original model also included sample source as a predictor variable, revealing a significant difference between CR-attorneys and snowball-attorneys, χ^2^(1) = 4.155, *p* = 0.042. That is, CR-attorneys (*M* = 0.88, *SD* = 0.33) showed slightly increased odds of recommending plea acceptance compared to snowball-attorneys (*M* = 0.81, *SD* = 0.40). However, when the model was conducted with and without the data from snowball-attorneys (*N* = 66), the results were not meaningfully different. Thus, we retained all participants in the final model to conserve statistical power. The estimated regression coefficients are listed in Table 1.

In the model, we first examined whether there was a significant interaction between defendant guilt status and potential trial sentence (Hypothesis #3). We analyze interactions before main effects because significant interactions qualify the interpretation of main effects, which may be misleading when interpreted alone. The results did not reveal an interaction for participants’ plea recommendations, χ^2^(2) = 1.071, *p* = 0.585. As such, the results did not support the hypothesis that defendant guilt status and potential trial sentence would interact to predict participants’ plea recommendations.

**Figure 1:**
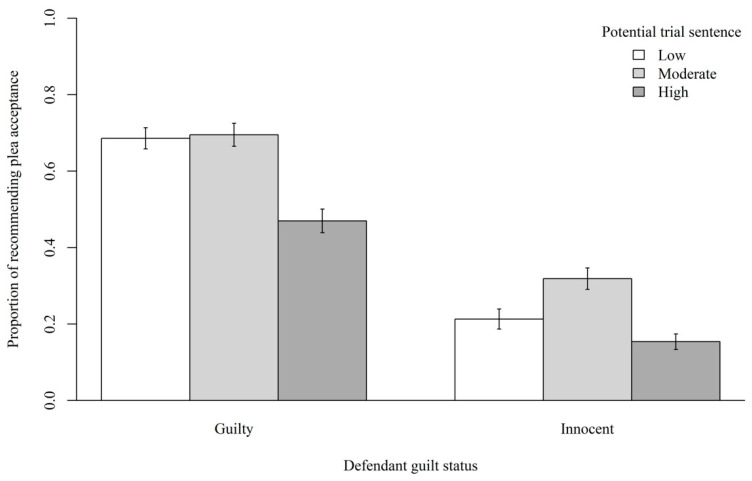
Proportions of Recommending Plea Acceptance in Each Condition. *Note*: Error bars indicate standard errors of the estimates.

We then examined whether there were any main effects (Hypotheses #1–2). The results revealed a significant main effect of defendant guilt status for participants’ plea recommendations, χ^2^(1) = 65.37, *p* < 0.001. Participants in the guilty (*M* = 0.62, *SD* = 0.49) defendant conditions showed increased odds of recommending plea acceptance compared to those in the innocent (*M* = 0.23, *SD* = 0.42) defendant conditions. The results also revealed a significant main effect of potential trial sentence for participants’ plea recommendations, χ^2^(2) = 13.52, *p* = 0.001. Participants in the high (*M* = 0.30, *SD* = 0.46) potential trial sentence conditions showed decreased odds of recommending plea acceptance compared to those in the moderate (*M* = 0.49, *SD* = 0.50) and low (*M* = 0.47, *SD* = 0.50) potential trial sentence conditions. Figure 1 displays the proportions of participants’ plea acceptance recommendations in each condition.

### 4.2. Willingness to Recommend Plea (WTRP)

To test participants’ willingness to recommend the plea (WTRP), we first explored descriptive statistics. Overall, participants’ average WTRP was 46%. When examining defendant guilt status, participants’ average WTRP was 59% in the guilty defendant conditions, compared to only 32% in the innocent defendant conditions. Regarding potential trial sentence, average WTRP rates were 51% in the low sentence conditions, 47% in the moderate sentence conditions, and 39% in the high sentence conditions.

Next, we tested Hypotheses #1–3 with a multiple linear regression model. The model included defendant guilt status, potential trial sentence, and their interaction term as predictor variables. The continuous outcome variable was participants’ WTRP (0–100%). The original model also included sample source as a predictor variable, revealing another significant difference between CR-attorneys and snowball-attorneys, *F*(1, 401) = 9.804, *p* = 0.033, *η*^2^*_p_* = 0.011. That is, CR-attorneys (*M* = 47.11, *SD* = 34.64) were more willing to recommend the plea compared to snowball-attorneys (*M* = 37.30, *SD* = 30.12). However, when the model was conducted with and without the data from snowball-attorneys (*N* = 66), the results were not meaningfully different. Thus, we retained all participants in the final model to conserve statistical power. The estimated regression coefficients are listed in Table 2.

We first examined whether there was a significant interaction between defendant guilt status and potential trial sentence (Hypothesis #3). The results did not reveal a significant interaction for participants’ WTRP, *F*(2, 397) = 7.284, *p* = 0.334, *η*^2^*_p_* = 0.002. As such, the results did not support the hypothesis that defendant guilt status and potential trial sentence would interact to predict participants’ WTRP.

**Figure 2:**
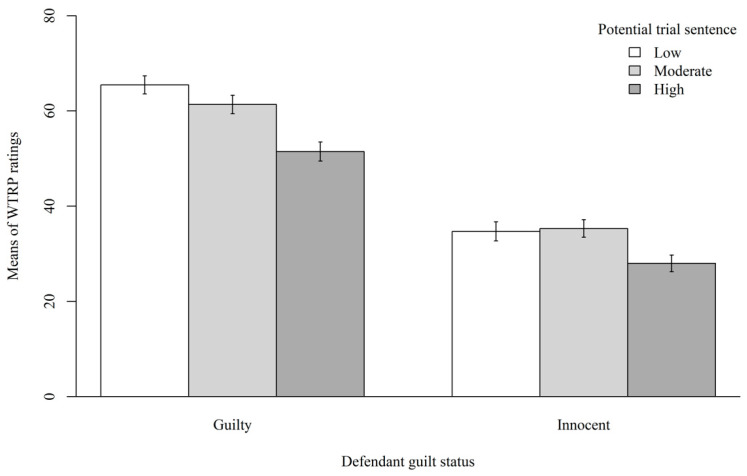
Means of WTRP in Each Condition. *Note*: Error bars indicate standard errors of the estimates.

We then examined whether there were any main effects (Hypotheses #1–2). The results revealed a significant main effect of defendant guilt status for participants’ WTRP, *F*(1, 397) = 5.646, *p* < 0.001, *η*^2^*_p_* = 0.156. Participants were more willing to recommend the plea in the guilty (*M* = 59.49, *SD* = 31.80) defendant conditions than innocent (*M* = 32.39, *SD* = 30.88) defendant conditions, *d* = 0.865. The results also revealed a significant main effect of potential trial sentence for participants’ WTRP, *F*(2, 397) = 2.623, *p* = 0.013, *η*^2^*_p_* = 0.022. Participants were more willing to recommend the plea in the low (*M* = 51.14, *SD* = 34.85) potential trial sentence conditions than moderate (*M* = 47.32, *SD* = 32.81, *p* = 0.455, *d* = 0.113) and high (*M* = 38.75, *SD* = 33.63, *p* = 0.009, *d* = 0.254) potential trial sentence conditions. Figure 2 displays the means of participants’ WTRP in each condition.

### 4.3. Maximum Plea Sentences

To test the maximum plea sentences participants would accept, we first explored descriptive statistics. Overall, participants’ average maximum acceptable plea sentence was 26 months. When examining defendant guilt status, participants’ average maximum acceptable plea sentence was 34 months in the guilty defendant conditions, compared to only 18 months in the innocent defendant conditions. Regarding potential trial sentence, average maximum acceptable plea sentence was 8 months in the low sentence conditions, 14 months in the moderate sentence conditions, and 53 months in the high sentence conditions.

Next, we tested Hypotheses #1–3 with a multiple linear regression model. The model included defendant guilt status, potential trial sentence, and their interaction term as predictor variables. The continuous outcome variable was participants’ maximum acceptable plea sentence (i.e., years/months). The original model also included sample source as a predictor variable, which did not reveal a difference between CR-attorneys and snowball-attorneys, *F*(1, 401) = 4.688, *p* = 0.408, *η*^2^*_p_* = 0.002. Thus, we retained all participants in the final model. The estimated regression coefficients are listed in Table 3.

**Figure 3:**
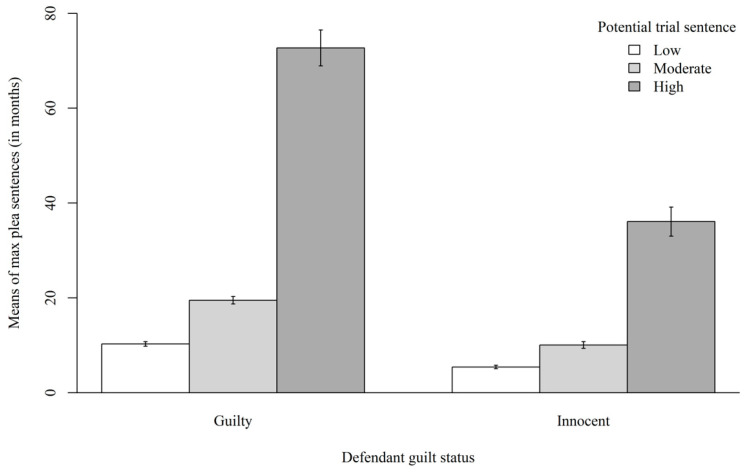
Means of Maximum Plea Sentences in Each Condition. *Note*: Error bars indicate standard errors of the estimates.

We first examined whether there was a significant interaction between defendant guilt status and potential trial sentence (Hypothesis #3). The results revealed a significant interaction for participants’ maximum plea sentences, *F*(2, 397) = 34.42, *p* < 0.001, *η*^2^*_p_* = 0.039. Because of the significant interaction, we probed the simple effects. When the defendant was guilty, potential trial sentence significantly affected participants’ maximum plea sentence such that they would accept longer sentences in the high potential trial sentence conditions than moderate and low potential trial sentence conditions, *t*(400) = 10.0, *p* < 0.001. Similarly, when the defendant was innocent, potential trial sentence significantly affected participants’ maximum plea sentence such that they would accept longer sentences in the high potential trial sentence conditions than moderate and low potential trial sentence conditions, but to a lesser degree, *t*(400) = 5.12, *p* < 0.001. Therefore, the effect of potential trial sentence was moderated by defendant guilt status for participants’ maximum acceptable plea sentences. Figure 3 displays the means of participants’ maximum plea sentences in each condition.

We then examined whether there were any main effects (Hypotheses #1–2). The results did not reveal a significant main effect of defendant guilt status for participants’ maximum plea sentences, *F*(1, 397) = 0.785, *p* = 0.433, *η*^2^*_p_* = 0.059. As such, the results did not support the hypothesis that participants would accept longer sentences in the guilty defendant conditions than innocent defendant conditions. However, the results revealed a significant main effect of potential trial sentence for participants’ maximum plea sentences, *F*(2, 397) = 10.29, *p* < 0.001, *η*^2^*_p_* = 0.255. Participants would accept longer sentences in the high (*M* = 52.88, *SD* = 60.28) potential trial sentence conditions than moderate (*M* = 14.42, *SD* = 12.81, *p* = 0.141, *d* = 0.173) and low (*M* = 8.04, *SD* = 7.38, *p* < 0.001, *d* = 1.039) potential trial sentence conditions.

## 5. Discussion

The current research investigated the combined effects of defendant guilt status and potential trial sentence on attorneys’ plea-related recommendations. We examined both main and interactive effects of these variables across three outcomes: plea recommendations (accept or reject), willingness to recommend the plea (WTRP), and maximum acceptable plea sentences (years/months). Overall, the results revealed a consistent influence of potential trial sentence across all dependent variables. Defendant guilt status appeared to affect attorneys’ plea recommendations and WTRP but had less influence on their maximum acceptable plea sentences. However, this pattern was qualified by an interaction, as the effect of sentence severity varied depending on the defendant’s guilt or innocence. The following discussion examines how these findings align—or do not align—with the predictions of PT’s utility function.

Our findings provide some support for PT’s utility function. When the defendant was guilty, attorneys were more likely to recommend plea acceptance and expressed greater willingness to do so. This pattern suggests risk-averse behavior in the guilty conditions, where accepting the plea may be seen as a safer resolution to avoid harsher trial outcomes. In contrast, fewer plea acceptance recommendations and less willingness to recommend the plea in the innocent conditions may reflect attorneys’ reluctance to endorse punishment for a client they believe did not commit the crime. This pattern may reflect a greater inclination toward risk-seeking behavior, as attorneys favored proceeding to trial in hopes of securing a full acquittal. These findings suggest that attorneys’ plea decisions are shaped not only by legal strategy, but also by perceived fairness tied to the defendant’s culpability. Though, it is also possible that attorneys’ knowledge of the defendant’s culpability influenced their perceptions of conviction probability.

Our findings also provide support for PT’s utility function in how potential trial sentence influenced attorneys’ maximum acceptable plea sentences. Plea acceptance recommendations and WTRP actually dropped under high sentence conditions, likely due to our high sentence condition simulating an overcharging scenario. However, attorneys accepted longer sentences as potential trial sentences increased. This pattern aligns with PT’s utility function in that larger potential losses can enhance risk aversion (making larger penalties more acceptable), but only up to a point that attorneys perceive as reasonable. Beyond that point, the excessive penalty may instead prompt more risk-seeking behavior, with attorneys favoring trial over an unjust plea deal. Future research could explore how perceived fairness or legitimacy impact attorneys’ plea decisions in high-stakes or overcharging scenarios.

Lastly, our findings support PT’s utility function in how defendant guilt status interacted with potential trial sentence to predict attorneys’ maximum acceptable plea sentences, but not their plea recommendations or WTRP. When the defendant was guilty, accepting a longer sentence may have reflected risk-averse behavior (i.e., choosing a certain but lesser penalty over the uncertainty and potential severity of trial). As potential trial sentences increased, attorneys accepted correspondingly longer plea sentences. In contrast, when the defendant was innocent, recommending trial may have reflected risk-seeking behavior (i.e., rejecting a certain punishment in favor of the uncertain possibility of acquittal). Thus, higher potential trial sentences led attorneys to accept longer plea sentences for both guilty and innocent defendants. However, this effect was stronger for guilty than for innocent defendants. These patterns suggest that both perceived culpability and sentence severity shape the boundaries of what attorneys view as an acceptable plea outcome. Given that attorneys’ plea decision-making is influenced by many factors, the following sections discuss the ethical, legal, and psychological considerations of this research.

### 5.1. Ethical Considerations

An important ethical consideration of our findings is how attorneys recommended plea acceptance more frequently and expressed greater willingness to do so when the defendant was guilty rather than innocent. While this pattern aligns with PT’s utility function, it may also reflect moral and professional norms within the legal community. Specifically, many attorneys may view advising an innocent client to plead guilty as fundamentally unjust, even if it offers strategic advantages. This reluctance likely stems from a broader commitment to the ethical principle that no individual should be punished for a crime they did not commit—a principle that, in practice, can outweigh risk-minimization strategies. One attorney in our sample put it succinctly: “It comes down to weighing the length of the prison term at trial versus the plea, while also weighing guilt and innocence,” capturing the conflict between strategic advocacy and ethical integrity.

This interpretation aligns with experimental work from Tor et al. (2010), who found that perceptions of unfairness reduced defendants’ willingness to accept plea offers, even when they were objectively advantageous. Applied to attorneys, this suggests reduced plea recommendations for innocent defendants may stem not just from legal strategy but also from moral discomfort with endorsing a plea in a situation perceived as unjust. Procedural justice theories further emphasize that attorneys’ fairness perceptions play a central role in how they advise clients (Bibas, 2016; O’Hear, 2008). That is, attorneys’ plea decision-making is shaped by their perceptions of factors such as the legitimacy of prosecutorial conduct, proportionality of potential trial outcomes, and integrity of the legal process. These perceptions influence not only how attorneys evaluate plea offers but also the strategies they employ during negotiations. As one attorney in our sample noted, “The value of a plea bargain depends largely on the reasonableness of the prosecutor,” underscoring how views of prosecutorial fairness can directly shape their plea decisions. Future research should investigate how moral reasoning, procedural fairness, and strategic risk assessment interact in attorneys’ plea decision-making.

### 5.2. Legal Considerations

An important legal consideration of our findings is how they intersect with landmark cases governing the voluntariness of guilty pleas, particularly when the potential trial sentence is high. These legal precedents suggest that large sentencing discounts and even threats of the death penalty do not constitute coercion in plea bargaining (*Bordenkircher v. Hayes*, 1978; *Brady v. United States*, 1970). Our results show that heightened trial penalties increased plea acceptance recommendations and maximum acceptable plea sentences. One attorney in our sample reflected on this dynamic, “Sometimes it is best to take a deal to avoid the risk of trial,” highlighting how the prospect of severe trial sentences can weigh heavily on the plea advice they provide. However, these patterns also raise questions about whether the legal precedents of plea bargaining account for pressures created by steep sentencing differentials, especially when such sentences approach what attorneys perceive as excessive or disproportionate. In this study, participants recognized that a 10-year plea sentence was too excessive to accept, but it still significantly influenced the maximum penalty they deemed acceptable. Legal assumptions embedded in plea bargaining often presume that decisions are the product of rational cost–benefit calculations, overlooking how contextual factors like sentence severity can influence decision-making beyond purely strategic reasoning (Helm, 2022; Helm et al., 2022).

In theory, plea decisions are framed as free choices between equally legitimate options, yet in practice, factors like steep sentencing differentials and prosecutorial discretion can heavily influence them. Our findings suggest that increasing potential trial sentences can shape both attorneys’ recommendations to clients and their perceptions of acceptable plea outcomes. These dynamics challenge the legal assumption that plea decisions result from rational cost–benefit calculations. In fact, research shows that large sentencing gaps can exert substantial coercive pressure, effectively penalizing the constitutional right to trial (McCoy, 2005; Wilford & Bornstein, 2023). Helm (2023) further notes that such pressures are often underestimated in legal doctrine, which overlooks how contextual factors alter plea decisions beyond strategic reasoning. As one attorney in our sample observed, “Plea bargaining by charging something really high and then negotiating down is ultimately a tool that the State uses to bully people into harsh sentences in cases where the evidence is shaky,” illustrating how prosecutorial overcharging and steep trial penalties can transform plea bargaining from a calculated choice into a pressured decision shaped by both practical and moral concerns. Future research should assess whether current legal assumptions adequately guard against the coercive potential of extreme sentencing disparities.

### 5.3. Psychological Considerations

An important psychological implication of our findings is that they not only align with predictions of the Shadow of the Trial (SoT) model but also extend understanding of how SoT factors shape attorney decision-making. SoT holds that plea decisions are primarily guided by conviction probability, potential trial sentence, plea sentence, and defendant guilt status (Bushway & Redlich, 2012; Bushway et al., 2014; Wilford et al., 2021a), and existing research on attorneys’ plea recommendations generally supports this view (Cardenas, 2023; Fessinger et al., 2024; Hellgren & Kassin, 2022; Henderson, 2021; Kramer et al., 2007). Our results demonstrated that potential trial sentence and defendant guilt status independently influenced attorneys’ plea recommendations and WTRP, similar to prior research on attorneys’ plea recommendations. But these factors also interacted to predict attorneys’ maximum acceptable plea sentences, such that higher potential trial sentences led to acceptance of longer plea sentences, with the effect being stronger for guilty rather than innocent defendants. This partial departure from SoT may reflect anchoring (Tversky & Kahneman, 1974), wherein trial sentences serve as reference points that shape judgments about acceptable plea lengths (Cardenas, 2023). One attorney in our sample explained, “Prosecutors seek to maximize potential sentences in order to scare people into pleading rather than relying on the trial process. There is often little actual judgment or concern, it is a numbers game for all concerned,” indicating that such practices may reinforce reliance on anchors rather than individualized case assessments. These reflections suggest that attorneys may rely on potential trial sentences as cognitive anchors, influencing whether they adopt risk-averse (accepting higher pleas to avoid trial) or risk-seeking strategies (pushing cases forward despite risks). Taken together, these findings reinforce the predictive value of SoT factors while adding nuance about how they may interact to shape attorneys’ risk preferences. A stronger empirical test of anchoring will be necessary to more precisely assess its role, but our results suggest that it represents a meaningful extension of the SoT model.

Another important psychological consideration of our findings comes from Fuzzy Trace Theory (FTT), which could account for why attorneys’ plea recommendations did not increase consistently with sentence severity. FTT suggests that decision-makers often rely on *gist* impressions (i.e., categorical evaluations such as “short”, “moderate,” or “very long”) rather than exact numerical differences (Zottoli et al., 2023). These gist-based judgments can guide plea decisions even in the face of substantial numerical differences, resulting in outcomes that would seem irrational from a purely economic perspective (Reyna et al., 2025). Our findings showed relatively small changes in plea recommendations and WTRP when the potential trial sentence increased from two to four years, indicating it may have been insufficient to produce a categorical change. However, when potential trial sentence jumped to 20 years in the overcharging scenario, plea acceptance recommendations declined. This may be because a 20-year sentence fell into a “very long” or “excessive” category, diminishing perceived benefit of the plea while heightening concerns about fairness and proportionality. Consistent with Zottoli et al. (2023), these findings indicate that attorneys’ plea decision-making may be driven more by categorical perceptions of sentence severity than by the precise numerical gap between trial and plea outcomes. As one attorney noted, “Twenty years for drug possession seems grossly excessive and I believe there is a high probability that, even if convicted, the defendant would not receive the maximum sentence”, capturing how perceived excessiveness can shift attitudes from risk-avoidance to risk-seeking. Future research should further examine how gist-based reasoning relates to attorneys’ perceptions and recommendations in overcharging contexts.

### 5.4. Implications for Attorneys’ Plea Decision-Making

The current findings have important implications for understanding how attorneys approach plea decision-making. Our results provide a different perspective on attorneys’ plea recommendations compared to prior work. For example, Edkins (2011) measured the sentence that attorneys believed they could secure for their client, whereas our outcome variable captured the maximum plea sentence attorneys would accept. This shifts the focus from estimating likely negotiation outcomes to identifying the range of plea terms attorneys consider acceptable. By focusing on acceptable rather than anticipated results, our findings highlight a different dimension of the plea decision-making process. Specifically, they suggest that attorneys’ plea recommendations may be influenced not only by legal strategy but also by risk attitudes shaped by defendant guilt and sentencing differentials. More broadly, they may also reflect a combination of factors, including perceptions of fairness and the practical realities of negotiation.

Another way our results differ from prior work involves the effect of defendant guilt status on attorneys’ plea decision-making. Attorneys were more likely to recommend plea acceptance when the defendant was guilty rather than innocent, consistent with previous research on defendants’ plea decisions (Dervan & Edkins, 2013; Redlich & Shteynberg, 2016; Wilford et al., 2021b). While this might seem inconsistent with research suggesting attorneys are more influenced by objective conviction probability than guilt status (Hellgren & Kassin, 2022), our simulation was designed to establish factual guilt. Therefore, it introduced minimal evidence, and the defendant’s claim of guilt or innocence were confirmed by a flashback scene revealing their guilt status. In this context, attorneys may have assumed that innocent defendants were less likely to be convicted, which in turn influenced both their willingness to recommend the plea and recommendations. However, defendant guilt status did not have a direct independent effect on maximum acceptable plea sentences. Although attorneys accepted lower maximum plea sentences for innocent defendants, this effect was moderated by the potential trial sentence. These patterns provide further context for understanding the factors that inform attorneys’ plea decision-making and how they may differ from extant research.

Taken together, these findings contribute to a more nuanced understanding of how case-specific factors shape attorneys’ plea recommendations. By examining the combined influence of defendant guilt status and potential trial sentence, our results illustrate how attorneys’ decision-making may shift depending on the context. Such insight is important for better understanding how large sentencing gaps and defendant guilt could create pressures within plea negotiations, potentially contributing to the coercive effects often attributed to substantial trial penalties.

### 5.5. Limitations and Future Directions

The current research has some limitations. First, we relied on a sample of practicing attorneys working in various domains (e.g., criminal defense, civil litigation, etc.) in the legal space. One-fifth of our sample (19%) consisted of criminal defense attorneys and prosecutors, while the remainder of the sample consisted of attorneys from other domains. Attorneys make up approximately 0.4% of the United States population, and only 1% of all attorneys practice criminal defense (American Bar Association, 2020). Because of this, criminal defense attorneys are extremely expensive and time-consuming to recruit. Thus, recruiting solely criminal defense attorneys was not feasible for this study. Now that these effects have been observed with practicing attorneys, future research should attempt to replicate these findings using a more specialized sample of criminal defense attorneys.

Second, our findings revealed significant effects of sample source for most dependent variables of interest. The sample of snowball-attorneys comprised mostly criminal defense attorneys, whereas the sample of CR-attorneys was more mixed in terms of legal specialization. The sample of snowball-attorneys was less likely to recommend plea acceptance and less willing to recommend the plea than CR-attorneys. Although we could not statistically compare criminal defense attorneys’ responses to those of other attorney types because of the limited sample size, we did examine descriptive statistics and found that criminal defense attorneys followed the same general patterns for plea recommendations, WTRP, and maximum acceptable plea sentences, but to a lesser degree. We suspect this may be because our study did not include the negotiation dynamics that typically occur during plea bargaining. This effect was reflected in the open-ended responses from snowball-attorneys. For instance, one criminal defense attorney stated, “I would negotiate further to get the jail time down, but the defendant is likely going to have to serve some custodial sentence”. Although plea negotiations were beyond the scope of this study, future research should explore how attorneys navigate the negotiation aspect of plea bargaining to better understand the strategies and considerations that influence their recommendations.

Third, while the simulation was pilot tested with legal professionals and statistically evaluated for sensitivity, some participants questioned the realism of the sentencing scenarios. Moreover, while the simulation framed sentencing as prosecutor-driven, judges ultimately determine sentences in practice. These aspects of the simulation may limit its ecological validity and the extent to which it captures actual attorney-client plea discussions. Relatedly, we found main effects of defendant guilt status and potential trial sentence on plea recommendations and WTRP, but no interactions between the two. This pattern may reflect that defendant guilt status shaped perceived conviction probability and trial sentence severity shaped perceived cost, with both factors exerting additive rather than interactive effects. Another possibility is that, despite additional testing, our measures were not sensitive enough to detect subtler interaction effects. Future research should refine vignettes and plea scenarios in collaboration with legal practitioners to better capture institutional norms and realistic sentencing dynamics, potentially through participatory design methods or additional rounds of expert feedback.

Fourth, the simulation assumed that the defendant disclosed their guilt status to the attorney, which may not reflect real-world interactions. In practice, defendants do not always share this information truthfully—or at all—with their attorneys. Even when they do, attorneys might not believe them (Wilford et al., 2025). Although we added an additional layer to the simulation to address this concern (i.e., a “flashback” scene that confirmed the defendant’s guilt status after the disclosure), this narrative device also departs from practice. Future studies should investigate how ambiguity around defendant guilt impacts attorney decision-making to better approximate legal uncertainty and professional ethics.

Finally, the simulation focused on a drug offense in which the defendant’s culpability was made explicitly clear. While this clarity helped standardize responses across participants, it also limits the generalizability of our findings. Attorney decision-making may differ significantly in cases involving other types of offenses (e.g., violent crimes, white-collar offenses), in situations where a defendant’s guilt is less certain (or a defendant is “partially innocent”; Cardenas et al., 2023), or when sentencing guidelines vary across jurisdictions (e.g., state vs. federal). Future research should build on these findings by varying case characteristics to better understand how these factors influence plea-related recommendations in a broader range of legal contexts.

## 6. Conclusions

This research examined how attorneys evaluate plea offers when defendant guilt status and potential trial sentence vary, providing evidence that aligns with PT’s utility function. Attorneys’ plea acceptance recommendations increased as potential trial sentences increased (given offers remained within acceptable bounds), and they accepted longer maximum plea sentences as trial penalties increased. Attorneys accepted shorter maximum plea sentences for innocent defendants, but this effect was influenced by the potential trial sentence. These findings show how large sentencing differentials can shape plea advice in ways that raise concerns about the fairness of plea bargaining, particularly when potential trial sentences become substantial. Excessively large trial penalties may set the tone for negotiation such that defense attorneys find higher penalties more acceptable, which raises questions about their compatibility with landmark rulings on the voluntariness of resulting guilty pleas. Such dynamics point to a deeper tension in the plea-bargaining process: larger trial penalties may heighten the risk that negotiated pleas reflect coercion rather than genuine choice.

## Figures and Tables

**Table 1:** Regression Coefficients for Predicting Plea Acceptance Recommendations.

Variable	*b*	*SE*	*p*	OR	95% CI
Defendant guilt status	−2.086	0.405	<0.001 ***	0.124	[0.056, 0.275]
Potential trial sentence Interaction	−0.901 0.503	0.357 0.569	0.011 * 0.376	0.406 1.654	[0.202, 0.817] [0.543, 5.041]

*Note*: *** indicates significance at *p* < 0.001 and * indicates significance at *p* < 0.05.

**Table 2:** Regression Coefficients for Predicting WTRP.

Variable	*b*	*SE*	*p*
Defendant guilt status	−30.77	5.449	<0.001 ***
Potential trial sentence Interaction	−14.00 7.284	5.337 7.534	0.013 * 0.334

*Note*: *** indicates significance at *p* < 0.001 and * indicates significance at *p* < 0.05.

**Table 3:** Regression Coefficients for Predicting Maximum Plea Sentences.

Variable	*b*	*SE*	*p*
Defendant guilt status	−4.857	6.191	0.433
Potential trial sentence Interaction	62.41 −31.77	6.064 8.560	<0.001 *** 0.000 **

*Note*: *** indicates significance at *p* < 0.001 and ** indicates significance at *p* < 0.01.

## Data Availability

The research materials and data are available on the Open Science Framework at https://osf.io/9bacx/?view_only=9037b93886df4d7a9bcbd176182522ac (accessed on 9 October 2025).

## Abbreviations

The following abbreviations are used in this manuscript:

CR	CloudResearch
NACDL	National Association of Criminal Defense Lawyers
PT	Prospect Theory
WTRP	Willingness to recommend plea

## Appendix A

**Table A1:** Descriptive Statistics for Participant Sample.

	CloudResearch Sample		Snowball Sample		Total	
	*N*	%	*N*	%	*N*	%
Gender						
Male	112	33.23	32	48.48	144	35.73
Female	219	64.99	32	48.48	251	62.28
Non-binary/Genderqueer/Other	6	1.78	2	3.03	8	1.99
Race/Ethnicity						
White or Caucasian	222	65.88	52	78.79	274	67.99
Black or African American	35	10.39	0	0.00	35	8.68
Native American or Alaska Native	0	0.00	0	0.00	0	0.00
Native Hawaiian or Other Pacific Islander	0	0.00	0	0.00	0	0.00
Asian	29	8.61	2	3.03	31	7.69
Middle Eastern or North African	4	1.19	1	1.52	5	1.24
Hispanic or Latino/a/x	21	6.23	4	6.06	25	6.20
Other	26	7.72	7	10.61	33	8.19
Region						
Northeast	74	21.96	3	4.55	77	19.11
South	138	40.95	20	30.30	158	39.21
Midwest	53	15.73	9	13.64	62	15.38
West	72	21.36	34	51.52	106	26.30
Territories and Commonwealths	0	0.00	0	0.00	0	0.00
Density						
Frontier	2	0.59	1	1.52	3	0.74
Rural	20	5.93	12	18.18	32	7.94
Suburban	130	38.58	10	15.15	140	34.74
Urban	181	53.71	39	59.09	220	54.59
Other	4	1.19	4	6.06	8	1.99

## Appendix B

Interactive Computer Simulation Script—Drug Possession

The simulation opens with the defendant drinking with a friend at a house party. The sequence pans to another avatar who sells drugs to the friend, who then places the drugs in her purse.

Following, the friend asks the defendant to hold her purse as the screen fades into a black background with white text on the screen:

“A few hours later…”

The defendant is sitting in the front seat of his vehicle and the sequence pans to his cellphone, which indicates that he has texted the friend to ask where she is. A police officer approaches the vehicle, who then finds the drugs in the friend’s purse in the passenger seat of the defendant’s vehicle as a black background fades into a courtroom.

PROSECUTOR:

- Good afternoon, my name is Mr. Clark and I will be prosecuting this case on behalf of the State, Your Honor. »

DISTRICT COURT JUDGE:

- Good afternoon. What is the nature of this case, Mr. Clark? »

PROSECUTOR:

- The defendant, Ethan Foster, is accused of drug possession occurring around 10pm on the 26th of June. »
- In accordance with State law, drug possession occurs when a person has custody of drugs that are recognized by statute as being illegal. »
- According to the police report, there were illegal drugs found in the defendant’s car. »
- There is a witness willing to testify that they saw the defendant take drugs from another person at a party. »
- This is a textbook drug possession, which is considered a serious felony punishable by significant fines and/or time in jail. »
- We request a court date be set by the State as soon as possible. »

DISTRICT COURT JUDGE:

- The defendant, Ethan Foster, is being charged with drug possession. »
- At this time, he will be held until counsel has been appointed. »
- It is so ordered. »

A black background fades into the defense attorney’s office.

- You have been assigned to represent the defendant, Ethan Foster, in this case. »

The defendant appears in the defense attorney’s office.

DEFENDANT:

- Thank you for representing me. I can’t believe this is happening. I think I remember the night Mr. Clark is referring to…»

Here, the simulation shows two different sequences depending on whether the defendant is guilty or innocent.

If innocent:

The flashback sequence demonstrates that the defendant was offered drugs at the house party and turned them down.

DEFENDANT:

- I know I was offered drugs at the party, but I didn’t actually take them. I know I am innocent. »

If guilty:

The flashback sequence demonstrates that the defendant was offered drugs at the house party and took them.

DEFENDANT:

- I was offered drugs at the party and I took them. I know I am guilty. »

A black background fades into the prosecutor’s office.

- The prosecutor in this case, Mr. Clark, is interested in seeing whether the case could be resolved without a trial. »

Here, the simulation shows three different sequences depending on whether the potential trial sentence is low, moderate, or high.

If low:

PROSECUTOR:

- If this case does go to trial, I will be seeking the maximum penalty of 2 years in jail. »
- However, if the defendant agrees to plead guilty now, I am prepared to recommend that the judge sentence him to 1 year instead of 2 years in jail. »

If moderate:

PROSECUTOR:

- If this case does go to trial, I will be seeking the maximum penalty of 4 years in jail. »
- However, if the defendant agrees to plead guilty now, I am prepared to recommend that the judge sentence him to 2 years instead of 4 years in jail. »

If high:

PROSECUTOR:

- If this case does go to trial, I will be seeking the maximum penalty of 20 years in prison. »
- However, if the defendant agrees to plead guilty now, I am prepared to recommend that the judge sentence him to 10 years instead of 20 years in prison. »

The attorney-participant is then redirected back to Qualtrics to complete the survey.
